# Medically Related Post-traumatic Stress in Children and Adolescents with Congenital Heart Defects

**DOI:** 10.3389/fped.2017.00020

**Published:** 2017-02-13

**Authors:** Maya G. Meentken, Ingrid M. van Beynum, Jeroen S. Legerstee, Willem A. Helbing, Elisabeth M. W. J. Utens

**Affiliations:** ^1^Department of Child and Adolescent Psychiatry/Psychology, Erasmus MC – Sophia Children’s Hospital, Rotterdam, Netherlands; ^2^Division of Cardiology, Department of Pediatrics, Erasmus MC – Sophia Children’s Hospital, Rotterdam, Netherlands; ^3^Research Institute of Child Development and Education, University of Amsterdam, Amsterdam, Netherlands; ^4^Academic Center for Child Psychiatry the Bascule/Department Child and Adolescent Psychiatry, Academic Medical Center, Amsterdam, Netherlands

**Keywords:** congenital heart defect, post-traumatic stress, PTSD, children, adolescents

## Abstract

Children and adolescents with a congenital heart defect (ConHD) frequently undergo painful or frightening medical procedures and hospitalizations. They often need multiple invasive procedures at a very young age and require regular checkups during their entire life. From other pediatric populations, it is known that these kinds of experiences can result in acute stress reactions and even in post-traumatic stress disorder (PTSD) in the long-term. PTSD and also subthreshold PTSD can lead to serious (psychosocial) impairment. However, limited information is available about PTSD in children with ConHD. Therefore, the aim of this review is to provide a summary of the current literature on post-traumatic stress (PTS) in children and adolescents with ConHD describing the prevalence of PTSD and its predictors/correlates. This review indicates that a range of 12–31% of children undergoing cardiac surgery develop PTSD. A range of 12–14% shows elevated post-traumatic stress symptoms (PTSS). These findings are comparable to those of hospitalized children without ConHD. Noteworthy, most studies used varying self-report questionnaires to measure PTSD and only one study used a semistructured interview. Although all studies point in the same direction of elevated PTSD and PTSS, systematic research is necessary to be able to draw firm conclusions. At present, as far as we know, in most clinics treating patients with ConHD, there is no regular screening for PTS in children with ConHD. In the reviewed literature, there is strong consensus that screening for PTSS and (preventive) psychological care for children and adolescents with ConHD is urgently needed.

## Introduction

Medical events are often experienced as stressful and frightening. Especially in young patients, this can be overwhelming. Medical procedures and treatments often cause pain, fear, a feeling of helplessness, and may give a sense of life threat (1, 2). Furthermore, such procedures can challenge the beliefs of youngsters about the world as a safe place and can give rise to uncertainty about the future. Therefore, it is normal and understandable that some kind of postoperative stress occurs after the experience of such an event. The majority of young patients and their parents can handle this stress quite well (2, 3). However, some develop persistent traumatic stress reactions such as post-traumatic stress disorder (PTSD). This, in turn, can have a negative influence on medical adherence and consequently morbidity or even mortality and can lead to an increase in health care service use (4, 5). Furthermore, PTSD is associated with rehospitalizations, worse sleep quality, and impaired quality of life (6–8).

Children and adolescents with congenital heart defects (ConHDs) often undergo various invasive medical procedures at a very young age and some need lifelong checkups at the hospital and re-interventions (9). Therefore, children with ConHD seem to have a heightened risk for developing PTSD. Considering this and the impact that PTSD can have on medical and psychosocial functioning, research into this topic should be a priority. However, research regarding PTSD in youngsters mostly has focused on abuse, violence, accidents, and natural disasters (10). Disproportionately, few studies have looked into traumatized children and adolescents with ConHD.

This review aims to give an overview of what is known in the field of medically related post-traumatic stress (PTS) in children and adolescents with congenital heart disease and suggests future directions. To find all relevant articles, a multi-database search was done with support from the Biomedical Information Specialist of the Medical Library of the Erasmus MC. The databases used were Embase, Medline, PsychInfo, Web of Science, Scopus, and Google Scholar. The search was limited to English language articles published from the year 2000 onward. Keywords included in the search were posttraumatic stress disorder and congenital heart disease (with a variation of corresponding terms).

## Overview of Definitions of PTSD in Literature

In researching literature, different terms and abbreviations are used for posttraumatic stress (disorder). This variability makes it difficult to compare results across studies. In the following, all relevant terms and abbreviations are shortly discussed to highlight the differences. We suggest that future studies should use similar terms to make results more comparable across studies.

### Post-traumatic Stress (PTS)

The word “trauma” is often linked to physical injury. However, it can also refer to psychological injury or pain (11). Immediately after the experience of an unpleasant or stressful event, people may express unusual physical and emotional reactions. This acute distress in response to a traumatic event is called PTS. It is considered a normal and often adaptive response (12, 13). Stress reactions enable people to react directly to threatening situations and most people can return to a normal emotional state without help from professionals after such an event (14).

### Post-traumatic Stress Symptoms (PTSS)

The wide range of distressing physical and emotional reactions is sometimes also referred to as “symptoms” (15). PTSS is the term given to symptoms that can be experienced after a traumatic event. These symptoms include flashbacks, bodily sensations (e.g., sweating), avoidance of trauma-related aspects, emotional numbing, negative feelings, trouble with sleeping, anger, attention problems, hypervigilance, and others (16). As mentioned earlier, people are often confronted with some of these complaints after the experience of a traumatic event. However, the presence of some PTSS symptoms does not automatically lead to significant long-term impairment and must not be confused with a PTSD.

### Post-traumatic Stress Disorder (PTSD)

Fortunately, most people do not experience long-term negative reactions after a stressful event but cope with the distress in an adaptive way. However, some develop persistent traumatic stress reactions, such as PTSD. When we speak of PTSD, a specific constellation of PTSS is present in a persistent and significantly distressing way.

In the recent fifth edition of the diagnostic and statistical manual of mental health (DSM-V), the definition of PTSD has been considerably changed as compared to the formerly used DSM-IV. Yet, most psychological diagnostic and screening instruments (used in scientific research) are based on the DSM-IV criteria (17). Therefore, both will be addressed in the following.

#### PTSD in the DSM-IV

According to the DSM-IV, there are 17 PTSS which are grouped into three clusters: re-experience (cluster B), avoidance (cluster C) of the traumatic event, and increased arousal (cluster D) (18). To meet the diagnostic threshold for PTSD of the DSM-IV, individuals must experience at least one symptom of cluster B, three symptoms of cluster C, and two symptoms of cluster D in reaction to a traumatic event for more than a month.

#### PTSD in the DSM-V

The DSM-V lists 20 symptoms and divides them into 4 clusters instead of 3. They are called intrusion (cluster B), avoidance (cluster C), negative alterations in cognition and mood (cluster D), and alterations in arousal and reactivity (cluster E) (16). Compared to the DSM-IV, the number of symptoms that must be present for a diagnosis did not change, but the distribution over the different clusters did, as the individual must experience at least one cluster B, one cluster C, two cluster D, and two cluster E symptoms for more than a month. Besides, the DSM-V introduced a PTSD subtype for children 6 years and younger. The major change for preschool children is that in order to obtain a PTSD diagnosis only one symptom in either the “avoidance” or the “negative alterations in cognition and mood cluster” is needed.

### Subthreshold PTSD

Individuals can suffer from various PTSS without completely meeting all criteria for a PTSD. When this is the case, the literature speaks of “subthreshold,” “partial,” “subclinical,” or “subsyndromal” PTSD (19). Some authors also refer to elevated PTSS (13, 20). We suggest to use the term subthreshold PTSD as it refers best to patients who do not meet full PTSD criteria, and this term is also preferred by the World Health Organization (WHO) (21). Patients with subthreshold PTSD suffer from several PTSS but show too few symptoms to obtain a clinical diagnosis of PTSD (22). In literature, there is no strong consensus about a precise definition of subthreshold PTSD (19). Clinicians do not agree about the number of symptoms that must be experienced and to what extent all clusters must be present in order to determine the diagnosis of subthreshold PTSD. Therefore, there is a lot of variation in definition and nomenclature of this variable throughout the literature.

However, all definitions agree that even the presence of subthreshold symptoms can lead to serious impairment of everyday functioning and must not be ignored (22, 23). Unfortunately, subthreshold PTSD is not part of any official classification and is likely to be under-diagnosed. To improve the comparability of scientific findings regarding subthreshold PTSD, the WHO introduced the following definition: meeting two or three of the DSM-V criteria B–E (21). However, most screening questionnaires and diagnostic interviews used in the clinical practice and for research purposes still rely on the DSM-IV criteria, which makes it impossible to use the definition of the WHO. Therefore, updated versions of the instruments are highly needed. The three most frequently used DSM-IV definitions for subthreshold PTSD are (1) meeting criterion B plus C or D, (2) meeting two of the three criteria B, C, and D, and (3) having at least one symptom of each criterion (23). Despite the varying definitions across studies, prevalence rates were found to be most influenced by sample composition rather than definition (23).

### Pediatric Medical Traumatic Stress (PMTS)

Another term, which has been developed recently, is PMTS. PMTS refers to “a set of psychological and physiological responses of children and their families to pain, injury, serious illness, medical procedures, and invasive or frightening treatment experiences” (24). PMTS is related to subthreshold PTSD in the way that it represents a concept of PTSS when not all criteria for a PTSD are met. However, PMTS is limited to the pediatric setting. The underlying theory of PMTS offers a framework for comparable psychological responses in reaction to a variety of different pediatric injuries and illnesses (13).

## Diagnostic Instruments

About seven different types of validated instruments have been used to measure PTSS in children and adolescents in the pediatric setting (17). Three of them were used in scientific research into PTSD in children and adolescents with ConHD.

(1) The Diagnostic Interview Schedule for Children (DISC) (25) is a structured diagnostic instrument to screen for more than 30 developmental psychiatric diagnoses. The child version is suited for youngsters aged 9–17 years. There is also a parallel parent version for children aged 6–17 years. Furthermore, there is an interviewer-administered computer-assisted and paper-and-pencil version and a self-administered computerized audio version. The DISC contains 24 modules that can be administered individually. One of those modules is the anxiety disorder module that, among others, addresses the DSM-IV criteria of PTSD. Yet, no DSM-V version is available. The DISC has been shown to be a reliable and valid instrument (26, 27).
(2) The University of California at Los Angeles post-traumatic Stress Disorder Reaction Index (UCLA PTSD-RI) has a child, adolescent, and parent version. It can be administered verbally (questions are read out loud) or as a self-report (completed on paper). Norms for children and adolescents between 7 and 18 years are available. It was not designed to provide a PTSD diagnosis. The psychometric properties are good, and the UCLA PTSD-RI has been used widely (28, 29). A DSM-V version has been developed recently.
(3) The Impact of Event Scale-Revised (IES-R) (30) is a self-report instrument to measure subjective distress after a traumatic event. This questionnaire has not been developed to diagnose PTSD. However, research shows that it seems to be a solid instrument for the screening of PTS (31, 32). The IES-R has not yet been updated to the DSM-V criteria.

## PTS in Children and Adolescents with ConHD

In the early 1970s, Aisenberg et al. (33) for the first time raised attention to the psychological impact of cardiac catheterization and noted that especially young children showed post procedural emotional stress reactions. Despite the medical advances in pediatric cardiology and cardiac surgery over the last 30 years, a negative impact of those medical treatments on psychosocial functioning remains (34, 35). Since 2000, in total, five studies were published studying PTS in children and adolescents with a heart disease (see Table 1 for an overview). These few studies had heterogeneous samples, as only three studies included children and adolescents diagnosed with a *congenital* heart disease. Another study included children and adolescents with a genetic heart disease, and the remaining study did not mention the exact diagnoses of the participating patients:

**Table 1:** **Overview of studies into PTSD and PTSS in children and adolescents with ConHD**.

Reference	Sample size (*n*)	Age range (in years)	Sample population	Design	Instrument	PTSD (%)	PTSS (%)
Connolly et al. (36)	43	5–12	**Cardiac surgery**	Longitudinal follow-up study	DISC	12	12
Mintzer et al. (38)	104	12–20	Organ transplant (13 × heart)	Cross-sectional descriptive study	UCLA PTSD-RI	16	14
Toren and Horesh (37)	31	10–21	**CCHD**	Cross-sectional descriptive study	UCLA PTSD-RI	29	
Ingles et al. (40)	31	>15	ICD implant (for genetic heart disease)	Cross-sectional descriptive study	IES-R	31	50^a^
Evan et al. (39)	51	0–20	**Heart transplant**	Retrospective study	Retrospective chart review	0	34

*Studies including children with congenital heart disease are given in bold*.

*PTSD, post-traumatic stress disorder; PTSS, post-traumatic stress symptoms; CCHD, congenital cyanotic heart disease; ICD, implantable cardioverter defibrillator; DISC, Diagnostic Interview Schedule for Children; UCLA PTSD-RI, University of California at Los Angeles Post-traumatic Stress Disorder Reaction Index; IES-R, Impact of Event Scale-Revised; ConHD, congenital heart defect*.

*^a^PTSD rate in females*.

### PTS after Cardiac Surgery

Connolly et al. (36) studied 43 children between 5 and 12 years who underwent some type of cardiac surgery. No child had a diagnosis of PTSD pre-operatively. At postoperative assessment (4–8 weeks after discharge from the hospital), 12% of the children met diagnostic criteria for PTSD measured with the anxiety disorder module of the DISC. Both, the child and the parent versions of the DISC, were administered and scored jointly. It is stated that 12% of the sample showed PTSS. Furthermore, no follow-up assessment was done.

Toren and Horesh (37) studied PTSD in adolescents who had an operation for congenital cyanotic heart disease. Thirty-one adolescents between 10 and 21 years participated, of which 29.03% scored “full PTSD likely” on the adolescent version of the UCLA PTSD-RI. Interesting fact is that PTSS were measured 13.7 years (SD = 2.48) after cardiac surgery in this study. Thus, PTSS seemed to be present in adolescents with ConHD long after surgery.

### PTS after Transplantation

Mintzer et al. (38) studied 104 adolescent organ transplant recipients, of which 13 adolescents received a heart transplant. The adolescents were 12–20 years old. PTSD symptoms were measured with the adolescent version of the UCLA PTSD-RI. The authors categorized respondents as “full PTSD likely,” when PTSD criteria were met, and “partial PTSD likely,” when adolescents met criteria for two of the three DSM-IV symptom clusters. They found that 16.3% were “full PTSD likely” and an additional 14.4% were “partial PTSD likely.” The assessment took place 7.3 years (SD = 7.3) after transplantation surgery. No difference in PTSD symptom severity was found between the organ types (liver, heart, and kidney).

Evan et al. (39) did a retrospective chart review to look for PTSS in pediatric heart transplant recipients aged 0–20 years. They reviewed 51 consecutive patients (of which 12 were known with a ConHD) and checked the medical history for any PTSS; 34% were found to have PTSS (at least 1 PTSD symptom according to the DSM-IV) up to 1 year after transplantation. Presence of PTSS was even higher around surgery: 43% were found to have PTSS in the peritransplant period. No patient was reported to have a full PTSD. It must be noted that these findings are speculative as they do not rely on prospective data from validated instruments.

### PTS after ICD Implantation

Ninety patients (15 years and older, mean = 49 years, SD = 14) with a clinical diagnosis of a genetic heart disease and an ICD implant participated in the study of Ingles et al. (40). Only those who had experienced at least one ICD shock (*n* = 31) were asked to complete the IES-R. Thirty-one percent reported a score above the cutoff of 22, indicative of PTSD. Notably, 50% of the females who reported a shock showed PTSS.

### Predictors and Correlates

Connolly et al. (36) found that ICU length of stay (48 h and more) was the only predictor of postoperative PTSD symptoms in children aged 5–12 years who underwent cardiac surgery. The amount of hours spent at the ICU ranged between 0 and 1008 hours in this study. Cognitive level, negative reactivity and approach/withdrawal dimensions of temperament, and family support were no predictors of postoperative PTSD symptoms.

Mintzer et al. (38) found no association between any demographic (gender, ethnicity, age at interview) or illness-related (organ type, time since transplant, age at transplant) variables and PTSS severity. However, they found that illness onset (acute versus chronic) and medical complications in the past year (mild versus moderate/severe) did act as a significant predictor of PTSS when combined in the regression analysis. It is striking that adolescents with mild complications, rather than moderate/severe, had a higher chance of reporting PTSS. Furthermore, an acute onset also increased the risk for PTSS.

In the *general pediatric setting*, different factors predict the development of PTSS in children after injury (41):

– Child characteristics: prior internalizing (e.g., anxiety and depression) and externalizing (e.g., aggressive behavior) problems,
– Environmental characteristics: parental PTSS,
– Trauma-related factors: elevated heart rate immediately after injury and perceived severity of the event, and
– Cognitive processes: dysfunctional cognitive strategies/beliefs.

Remarkably, the *subjective* experience of life threat (trauma severity), rather than objective factors (mechanism, type, and severity of the injury), seems to contribute to the development of PTSS (41).

## PTS in Children and Adolescents without ConHD after Hospitalization

Since few studies focused on PTS in children and adolescents with ConHD, other pediatric populations can serve as an important reference framework. From other pediatric populations without ConHD, it is known that the experience of an injury or illness can lead to traumatic stress reactions in children and adolescents. Hospitalization, admission to the emergency department, entering intensive care, and undergoing medical interventions all heighten the risk for psychological problems alongside the evident physical complaints (41). Research shows that even mild to moderate physical injury leads to heightened PTSS (42). The PTSD prevalence in children undergoing admission to the pediatric intensive care unit (PICU) has been shown to be between 5 and 28% (43). Despite the overlapping medical context, it seems that PTSD prevalence rates differ between young patients with a (chronic) illness and those with an injury (2). Both illness and injury often result in invasive procedures and hospitalizations. In addition, however, children who suffer an injury were also confronted with some kind of accident that may have been a traumatic experience itself. This might explain why injured children and adolescents show higher rates of PTSD than ill children and adolescents. Furthermore, young patients rate the perceived trauma severity and/or life threat higher when injured, compared to children with an illness. However, this might also be due to differences in follow-up measurements. Across research, children who experienced an injury were followed up for a shorter time span. Nevertheless, it is recommended to disentangle the traumatic impact of illness and injury samples when studying PTSD.

## Conclusion and Clinical Implications

Only five studies have been found that focused on PTSD in children and adolescents with different heart diseases, of which one did not use standardized measurements. The four studies using standardized instruments to measure PTSD in children with a (congenital) heart disease found PTSD prevalence between 12 and 31% even up to many years after the traumatic experience. This is comparable to 11–21% found in adults with ConHD (44). Compared to a lifetime PTSD prevalence of 5% in the general adolescent population (13–18 years) (45), youngsters with ConHD show a clearly heightened risk for PTSD. Two of the four described articles that used standardized measurements also studied the prevalence of subthreshold PTSD and found a prevalence of 12–14% in children and adolescents with ConHD. This is comparable with the mean subthreshold PTSD prevalence of 14.7% found in a meta-analysis of Brancu et al. (23).

Methodological weaknesses of the studies described are use of small sample sizes, different time intervals in follow-up assessments, the use of different instruments, and single- versus multi-informant approaches. Moreover, it is uncertain to what extent selection bias influenced the results. Only five studies into PTSD were found regarding children with ConHD using very specific samples. This lends to limited generalizability to the overall pediatric ConHD population. However, results are comparable to outcomes in other pediatric medical populations (such as children in the PICU).

In summary, children and adolescents with ConHD have an elevated risk of developing PTSD. Given the fact that both PTSD and subthreshold PTSD lead to serious psychological and behavioral impairments and increased health-care use (23), it is astonishing that only very few studies investigated the prevalence, correlates, and impact of PTSD in children and adolescents with ConHD. Even more concerning is that no study has evaluated an evidence-based treatment in this pediatric population yet. For adults, it has been proven already that eye movement desensitization and reprocessing (EMDR) is an effective psychotherapeutic treatment to reduce (symptoms of) PTSD (46). A large randomized controlled trial into the effectiveness of EMDR for children and adolescents with ConHD is now being executed in the Erasmus MC – Sophia Children’s Hospital, Rotterdam, the Netherlands. The authors of this review recommend early screening of psychosocial problems in children with ConHD, given the fact that those children have a heightened risk of developing PTSS. If indicated, referral for psychosocial treatment (trauma-focused cognitive behavioral therapy or EMDR) should be arranged.

## Author Contributions

This manuscript was written by MM with close collaboration and contribution of EU, WH, IB, and JL.

## Conflict of Interest Statement

The authors declare that the research was conducted in the absence of any commercial or financial relationships that could be construed as a potential conflict of interest.
